# NLRP3 activation contributes to endothelin‐1‐induced erectile dysfunction

**DOI:** 10.1111/jcmm.17463

**Published:** 2022-12-14

**Authors:** Rafael Sobrano Fais, Rafael Menezes da Costa, Allan Carvalho Mendes, Fabíola Mestriner, Simon Gabriel Comerma‐Steffensen, Rita C. Tostes, Ulf Simonsen, Fernando Silva Carneiro

**Affiliations:** ^1^ Department of Pharmacology, Ribeirao Preto Medical School University of Sao Paulo Ribeirao Preto Brazil; ^2^ Division of Pulmonary, Critical Care, and Sleep Medicine National Jewish Health Denver Colorado USA; ^3^ Special Academy Unit of Health Sciences Federal University of Goias Goiânia Brazil; ^4^ Department of Biomedicine, Pulmonary and Cardiovascular Pharmacology Aarhus University Aarhus Denmark

**Keywords:** corpus cavernosum, erectile dysfunction, ET‐1, inflammation, NLRP3

## Abstract

In the present study, we hypothesized that endothelin (ET) receptors (ET_A_ and ET_B_) stimulation, through increased calcium and ROS formation, leads to *Nucleotide Oligomerization Domain*‐Like Receptor Family, Pyrin Domain *Containing 3* (NLRP3) activation. Intracavernosal pressure (ICP/MAP) was measured in C57BL/6 (WT) mice. Functional and immunoblotting assays were performed in corpora cavernosa (CC) strips from WT, NLRP3^−/−^ and caspase^−/−^ mice in the presence of ET‐1 (100 nM) and vehicle, MCC950, tiron, BAPTA AM, BQ123, or BQ788. ET‐1 reduced the ICP/MAP in WT mice, and MCC950 prevented the ET‐1 effect. ET‐1 decreased CC ACh‐, sodium nitroprusside (SNP)‐induced relaxation, and increased caspase‐1 expression. BQ123 an ET_A_ receptor antagonist reversed the effect. The ET_B_ receptor antagonist BQ788 also reversed ET‐1 inhibition of ACh and SNP relaxation. Additionally, tiron, BAPTA AM, and NLRP3 genetic deletion prevented the ET‐1‐induced loss of ACh and SNP relaxation. Moreover, BQ123 diminished CC caspase‐1 expression, while BQ788 increased caspase‐1 and IL‐1β levels in a concentration‐dependent manner (100 nM–10 μM). Furthermore, tiron and BAPTA AM prevented ET‐1‐induced increase in caspase‐1. In addition, BAPTA AM blocked ET‐1‐induced ROS generation. In conclusion, ET‐1‐induced erectile dysfunction depends on ET_A_‐ and ET_B_‐mediated activation of NLRP3 in mouse CC via Ca^2+^‐dependent ROS generation.

## 1 INTRODUCTION

Endothelin‐1 (ET‐1) is a peptide that produces potent, slow, and sustained contractions.^1^ ET‐1, mainly through ET type A receptor (ET_A_), is often associated with the genesis and development of cardiovascular diseases,^2^, ^3^, ^4^ including erectile dysfunction (ED).^5^, ^6^

Experimental rat models of alcoholism and diabetes exhibit increased corpora cavernosa (CC) ET_A_ and ET_B_ expression.^7^ Furthermore, ET‐1, via ET_A_ and ET_B_, mediates oxidative stress‐induced endothelial dysfunction in penile arteries from insulin‐resistant obese Zucker rats.^8^ Thus, ET‐1 antagonists have pronounced protective effects against ED in animals. Unfortunately, ET‐1 antagonists have so far failed to treat ED in clinical trials.^5^

Endothelin‐1 stimulates the release of inflammatory mediators, such as IL‐1β, IL‐6, and TNF‐α.^9^, ^10^ In addition, ET‐1 also stimulates proinflammatory transcription factors, such as NF‐κB in the retina, kidney, and heart^11^ and activator protein 1 (AP‐1) in mesangial cells.^11^, ^12^ Moreover, ET‐1 increases the expression of vascular cell adhesion molecule (VCAM‐1), endothelial selectin (E‐selectin), and increases the interactions of endothelial and immune cells in deoxycorticosterone acetate (DOCA)/salt hypertensive rats by ET_A_ activation.^13^

Endothelin‐1 increases reactive oxygen species (ROS) generation, which positively modulates inflammatory processes in the liver,^14^ lungs,^15^ and blood vessels^16^ of rats. These effects are mainly due to NF‐κB, cyclooxygenase (COX), and NAD(P)H oxidase activation.

The vasocontractile and proliferative effects of ET‐1 are highly dependent on Ca^2+^ influx.^17^, ^18^ ET_A_ stimulates contraction by increasing Ca^2+^ signalling. Increased Ca^2+^ via ET_B_ receptors also contribute to contractions in penile arteries of obese Zucker rats.^19^ Moreover, ET‐1‐induced Ca^2+^ increases IL‐6 release in the pulmonary artery,^17^ airway smooth muscle cells,^20^ and monocytes.^21^ Additionally, ET‐1‐induced ROS generation is related to Ca^2+^ influx in pulmonary vascular smooth muscle,^22^ and mesangial cells.^23^ ET‐1 has several effects on the nerves, regulating Ca^2+^ influx and ROS generation, e.g., ET‐1 also regulates sympathetic activity via nerve growth factor in the heart^24^ and controls blood pressure via ET_B_ in sensory nerves.^25^

Endothelin‐1 leads to the activation of pattern recognition receptors (PRRs), such as Toll‐like receptors (TLRs) and NLRP (nucleotide‐binding oligomerization domain, leucine‐rich repeat and pyrin domain‐containing) inflammasomes.^26^ TLR4 induces activation of NF‐кB, which is the first signal for the NLRP3 inflammasome assembly.^27^ Another signal for NLRP3 activation involves pore‐forming proteins in the membrane that, by P2X channel activation, leads to Ca^2+^ influx,^28^, ^29^ ROS generation,^30^ and phagolysosomal or mitochondrial destabilization.^31^ These stimuli lead to NLRP3 inflammasome assembly, promoting caspase‐1 activation and IL‐1β and IL‐18 release in their mature form.^27^, ^31^, ^32^, ^33^, ^34^

Activation of the NLRP3 inflammasome decreases CC sensitivity to nitric oxide (NO) and endothelium‐dependent relaxation,^35^ processes that may be associated with NLRP3‐mediated vascular functional and structural damage.^36^, ^37^, ^38^, ^39^, ^40^ Considering that ET‐1 and the NLRP3 induce the release of inflammatory cytokines, which, in turn, modulate CC tonus,^6^, ^26^, ^27^, ^35^, ^41^ in the present study we hypothesized that endothelin receptor (ET_A_ and ET_B_) stimulation, through increased Ca^2+^ and ROS formation, leads to NLRP3 activation.

## 2 MATERIALS AND METHODS

### 2.1 Animals

Male, 10–12 weeks‐old C57BL/6 (WT), NLRP3^−/−^, and caspase‐1/11^−/−^ mice (~25 g) were housed in a room with controlled temperature (20–22°C) and on light/dark cycles of 12 h with free access to standard chow and filtered water. All experimental animal protocols adhered to the ARRIVE and BJP guidelines,^42^ followed the National Council's regulations on Animal Experimental Control (CONCEA) guidelines, and were approved by the Ethics Committee on Animal Experimentation (CEUA n° 005/2015–1) of the Ribeirao Preto Medical School, University of Sao Paulo. All animal experiments performed at Aarhus University followed Danish legislation of animal use for scientific procedures as described in the ‘Animal Testing Act’ (Consolidation Act No. 726 of 9 September 1993 as amended by Act No. 1081 of 20 December 1995) and were approved by the Danish Animal Experiments Inspectorate (permission 2019‐15‐0201‐00009).

### 2.2 In vivo measurements of intracavernosal pressure and mean arterial pressure

The animals were anaesthetised with 2% isoflurane in 100% oxygen (2 L.min^−1^). The left carotid artery and right CC of each mouse were then cannulated for continuous monitoring of mean arterial pressure (MAP) and intracavernosal pressure (ICP), respectively. Finally, the cavernosal nerve (CVN) was electrically stimulated with silver electrodes at 5 V, 1 ms pulse width, the frequency at 16 Hz, for 60 s (s) to induce changes in ICP and obtain the maximum (max) ICP/MAP ratio. Subsequently, the CVN was stimulated at the same parameters at frequencies varying from 2 to 8 Hz to determine the 50% (submaximal) of ICP/MAP for each animal.

Then, ET‐1 (0.2492 μg.Kg^−1^), MCC950 (0.42646 μg.Kg^−1^), or vehicle was injected in the intracavernosal tissue. A new submaximal stimulation was performed at 3, 10, 20 and 30 min (min), and a maximal stimulation at 25 min. A second intracavernosal administration of ET‐1 (0.2492 μg.kg^−1^) or vehicle was performed, and the submaximal stimulation was performed at 3, 10, 20 and 30 min, and maximal at 25 min. During the stimulation, these animals were maintained anaesthetised with isoflurane 1% in 100% oxygen (2 L/min).

### 2.3 Animal euthanasia

Mice were gently placed in a transparent chamber (7.5″ wide × 11.5″deep × 5″ high), and CO_2_ euthanasia was performed (2.0–5.0 CO_2_ L.min^−1^). After this procedure, the CC was isolated, split into two strips, and used for cavernosal reactivity or immunoblotting assays.

### 2.4 Cavernosal tissue reactivity

The cavernosal strips were isolated and mounted in 5 ml‐myograph chambers (Danish Myo Technology) containing buffer, which were continuously bubbled with a mixture of 95% O_2_ and 5% CO_2_ and maintained at 37°C. The tissues were stretched to a resting force of 2.5 mN and allowed to equilibrate for 60 min. Changes in isometric force were recorded using a PowerLab/8SP data acquisition system (Chart software, version 5.2; ADInstruments, Colorado Springs, CO). At the end of the equilibration period, phenylephrine (1 μM) was added to the organ baths to verify the contractile ability of the preparations. The CC strips were divided into five groups: 1) WT CC strips incubated with MCC950 (1 μM) or vehicle for 30 min, followed by ET‐1 or vehicle for 4 h, 2) WT CC strips incubated with tiron (100 μM) or vehicle for 30 min, followed by ET‐1 or vehicle for 4 h, 3) WT CC strips incubated with BAPTA AM (5 μg/ml) or vehicle for 30 min, followed by ET‐1 or vehicle for 4 h, 4) WT, NLRP3^−/−^ or caspase‐1/11^−/−^ CC strips incubated with ET‐1 or vehicle for 4 h, 5) BQ123 (1 μM) or BQ788 (1 μM) or vehicle for 30 min followed by ET‐1 or vehicle for 4 h. A solution containing a high concentration of potassium chloride (KCl, 120 mM) was added to the organ baths at the end of the protocols.

In CC strips contracted with phenylephrine (10 μM), relaxation responses were evaluated by performing cumulative concentration‐response curves for the endothelium‐dependent agonist, acetylcholine (ACh, 100 pM – 3 μM) and the NO donor, sodium nitroprusside (SNP, 10 pM – 100 μM)). Concentration‐response curves for SNP were performed after incubation with L‐NAME to prevent interference of basal NO production.

### 2.5 Western blot assay

Isolated CC, cleaned from surrounding fat tissue, were divided into five groups: group 1 – WT CC strips incubated with MCC950 or vehicle for 30 min followed by ET‐1 or vehicle for 4 h; group 2 – WT CC strips incubated with tiron or vehicle for 30 min followed by ET‐1 or vehicle for 4 h; group 3 – WT CC strips incubated with BAPTA AM or vehicle for 30 min followed by ET‐1 or vehicle for 4 h; group 4 – WT or NLRP3^−/−^ CC strips incubated with ET‐1 or vehicle for 4 h; group 5 – BQ123 (100 nM, 1 and 10 μM) BQ788 (100 nM, 1, and 10 μM) or vehicle for 30 min followed by ET‐1 or vehicle for 4 h.

After the incubation, the CC strips were snap‐frozen in liquid nitrogen and homogenised in a lysis buffer [50 mM Tris/HCl, 150 mM NaCl, 1% Nonidet P40, 1 mM EDTA, 1 μg/ml leupeptin, 1 μg/ml pepstatin, 1 μg/ml aprotinin, 1 mM sodium orthovanadate, 1 mM phenylmethanesulfonyl fluoride (PMSF), and 1 mM sodium fluoride] (Fais et al., 2019). Protein concentration was determined by the Lowry assay. Spectra multicolour broad range protein ladder (10 to 260 KDa) was used as a protein standard. Aliquots with 30 μg of proteins were prepared and separated by electrophoresis at 100 V for 2 h at 4° C in 10% polyacrylamide gel (SDS‐PAGE) and transferred for 1 h to a nitrocellulose membrane at 100 V at 4° C. Gels were stained with Coomassie blue and membranes with Ponceau red 2% to demonstrate the transference efficiency. Nonspecific binding sites of the membrane to the primary antibodies were blocked with 5% bovine serum albumin (BSA) solution for 1 h at room temperature. The primary antibodies described below were incubated for 12 h at 4°C, and the secondary antibodies were incubated for 1 h at room temperature. Protein bands visualization was obtained by chemiluminescence after ECL reaction (Amersham ECL Prime Western Blotting Detection Reagent) and image capture performed on ImageQuant 350 gel imager (GE Healthcare). The densitometric quantification was performed by ImageJ® software. Membranes were stripped with Restore Western Blot Stripping Buffer (Thermo) for 45 min at 37° C. Caspase‐1 and IL‐1β proteins were blotted in the same membrane. Therefore, both proteins share the same α/β‐tubulin image band for quantification analysis as an internal control and for representative image. The following antibodies were used in the study: caspase‐1 (diluted 1:500, Imgenex IMG‐5028. RRID: a, IL‐1β [diluted 1:500, Santa Cruz Biotechnology (H‐153)‐SC‐7884, RRID: AB_2124476]. The α/β‐tubulin (diluted 1:5.000, Cell Signalling 2148S, RRID: AB_2288042) expression was used as an endogenous control for all proteins' normalization. Membranes were then incubated with the following secondary antibodies: goat anti‐mouse IgG H + L (AB6789, diluted 1:10.000, Abcam, RRID: AB_955439) and goat anti‐rabbit IgG H + L (AB6721, diluted 1:10.000, Abcam, RRID: AB_955447).

### 2.6 Immunohistochemistry

Reactive oxygen specie generation in CC strips was assessed by dihydroethidium (DHE), as previously described.^43^ Briefly, CC strips were embedded in a medium for frozen tissue specimens to ensure optimal cutting temperature (OCT™) and stored at −80°C. Fresh‐frozen specimens were cross‐sectioned at 5 μm thickness and placed on slides covered with poly‐(L‐lysine) solution. The tissue was loaded with DHE (2.5 × 10^−6^ M; 30 min at 37°C), a non‐selective dye for ROS detection prepared in phosphate buffer (0.1 M). Images were collected on a Zeiss microscope and analysed by measuring the mean optical density of the fluorescence in a computer system (Image J software) and normalized by the area. Results are expressed as raw data relative to the control group

### 2.7 Drugs and solutions

Physiological Krebs Henseleit buffer of the following composition was used: NaCl 130 mM, KCl 4.7 mM, KH_2_PO_4_ 1.18 mM, MgSO_4_.7H2O 1.17 mM, NaHCO_3_ 14.9 mM, EDTA 0.026 mM, CaCl_2_.2H_2_O 1.6 mM and D‐glucose 5.55 mM. A high concentration of potassium chloride buffer of the following composition was used: NaCl 14 mM, KCl 120 mM, KH_2_PO_4_ 1.18 mM, MgSO_4_.7H_2_O 1.17 mM, NaHCO3 14.9 mM, EDTA 0.026 mM, CaCl2.2H2O 1.6 mM and D‐glucose 5.55 mM. The incubations were performed with ET‐1 (1, 10 and 100 nM, Tocris 1160; diluted in 5% BSA + 95% deionized water), MCC950 [1 μM,^44^ Cayman Chemical 17,510; diluted in 5% DMSO and 95% deionized water], tiron (100 μM, Santa Cruz SC253699; diluted in deionized water), BAPTA AM [5 μg/ml,^29^ Tocris 2787; diluted in deionized water], lipopolysaccharide (LPS) (1 μg/ml; diluted in deionized water), adenosine 5‐triphosphate (ATP) (2 mM, Sigma‐Aldrich A6144; diluted in deionized water), BQ123 (100 nM, 1, 10 μM, Tocris 1188; diluted in deionized water), BQ788 (100 nM, 1, 10 μM, Tocris 1500; diluted in deionized water).

### 2.8 Statistical analysis

According to each analysis, data from Western blot, CC reactivity, and immunohistochemistry protocols were analysed by one‐ or two‐way anova and followed by Tukey post‐test. The in vivo results were analysed by repeated‐measures two‐way anova and followed by Tukey post‐test. Values of *p* < 0.05 were considered statistically significant. Relaxation responses were expressed as the percentage change from pre‐contraction induced by phenylephrine. Concentration‐effect curves were submitted to nonlinear regression analysis using the GraphPad Prism program (GraphPad Prism 6.0; GraphPad Software Inc.). Relaxant potency and maximal response were expressed as pEC_50_ (negative logarithm of molar concentration producing 50% of the maximal response) and E_max_ (maximal effect produced by the agonist). Statistical analysis of the E_max_ and pEC_50_ values was performed using nonlinear regression followed by Student's *t*‐test or one‐way anova.

## 3 RESULTS

### 3.1 Erectile responses

**FIGURE 1:**
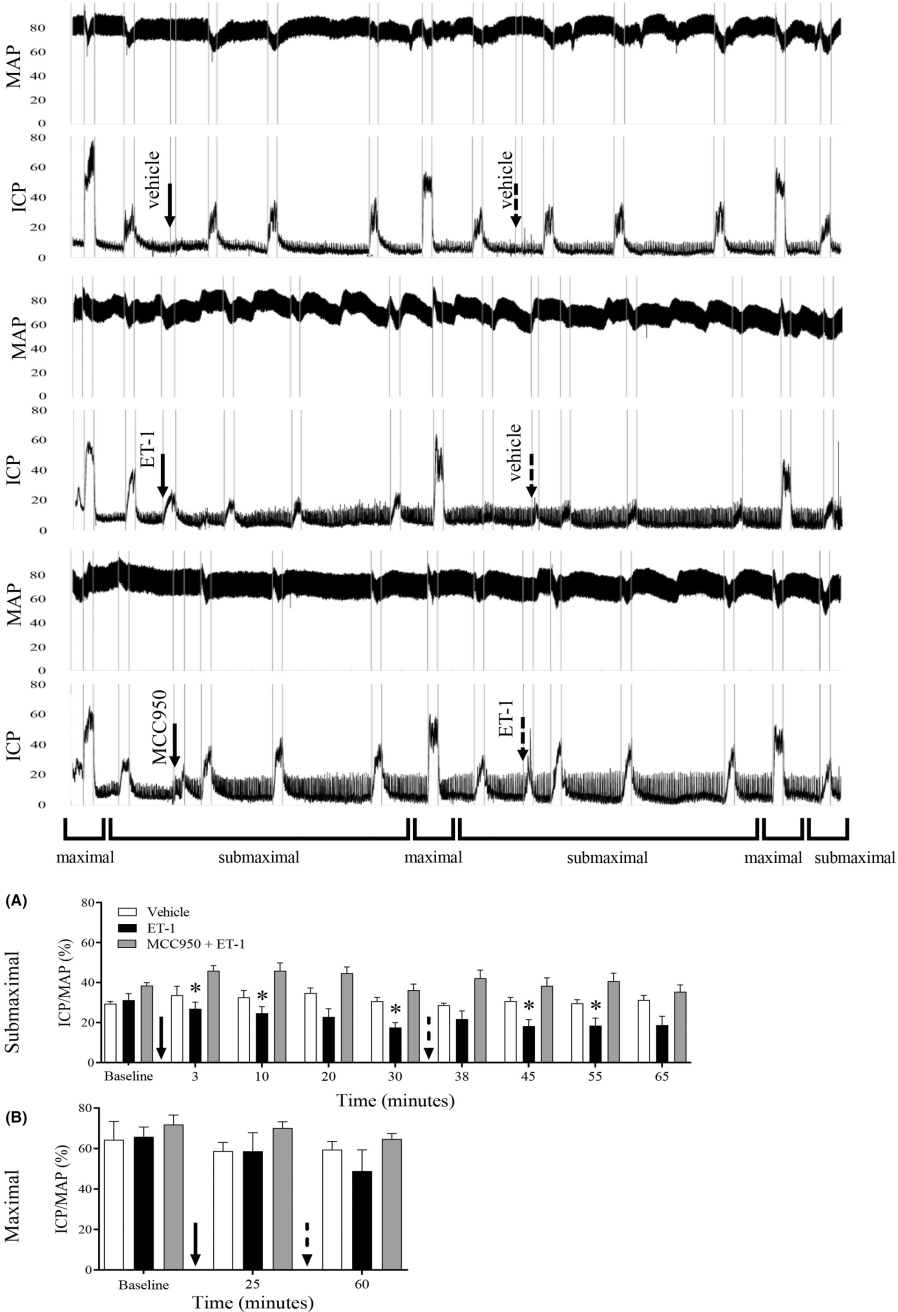
Effect of NLRP3 pharmacological inhibition of ICP/MAP ratio. The graph depicts the ICP/MAP ratio in response to cavernosal nerve stimulation: submaximal (A) (2, 4 or 8 Hz) at 3, 10, 20 and 30 min and maximal (B) (16 Hz) at 25 min after the injection of the vehicle followed by a new vehicle administration. Submaximal stimulation at 35, 38, 45, 55 and 65 min and maximal at 60 min (top tracings), ET‐1 followed by vehicle (middle tracings), MCC950 followed by ET‐1 (grey bars) (bottom traces) or vehicle (white bars) (top traces). Data represent the mean ± SEM of ICP/MAP values. Representative tracings showing changes in intracavernosal pressure and blood pressure in response to electrical stimulation of the cavernosal nerve. *p < 0.05 compared to vehicle group. *n* = 6. The comparison of each frequency value for the ICP/MAP was performed by repeated‐measures two‐way anova followed by Dunnet post‐test. ICP = intracavernosal pressure; MAP = mean arterial pressure. The maximal (16 Hz). The continuous arrow represents the first administration at 0 min and the dashed arrow represents the second administration at 35 min

In vivo stimulation of the cavernosal nerve in mice induced frequency‐dependent increase of ICP. ICP/MAP did not change after vehicle administration into the cavernosal tissue. All ICP/MAP responses remained unchanged along with the entire experimental protocol after vehicle injection (65 min) (Figure 1A,B).

Intracavernosal administration of ET‐1 reduced ICP/MAP responses to submaximal stimuli after 3, 10 and 30 min. The effect of ET‐1 was only washed out 3 min after intracavernosal administration of vehicle (at 38 min), and ET‐1 effects persisted throughout the entire experiment as observed at 45 and 55 min after its initial injection (Figure **1** **A**). ET‐1 did not change ICP/MAP responses evoked by maximal stimuli (Figure 1B).

The intracavernosal administration of MCC950 did not alter ICP/MAP response to submaximal (Figure 1A) or maximal (Figure 1B) stimulation. MCC950 completely abolished the effect of ET‐1 in ICP/MAP responses (Figure 1A,B).

### 3.2 Effect of endothelin‐1 and NLRP3 inhibition on ACh‐ and SNP‐induced relaxation

**FIGURE 2:**
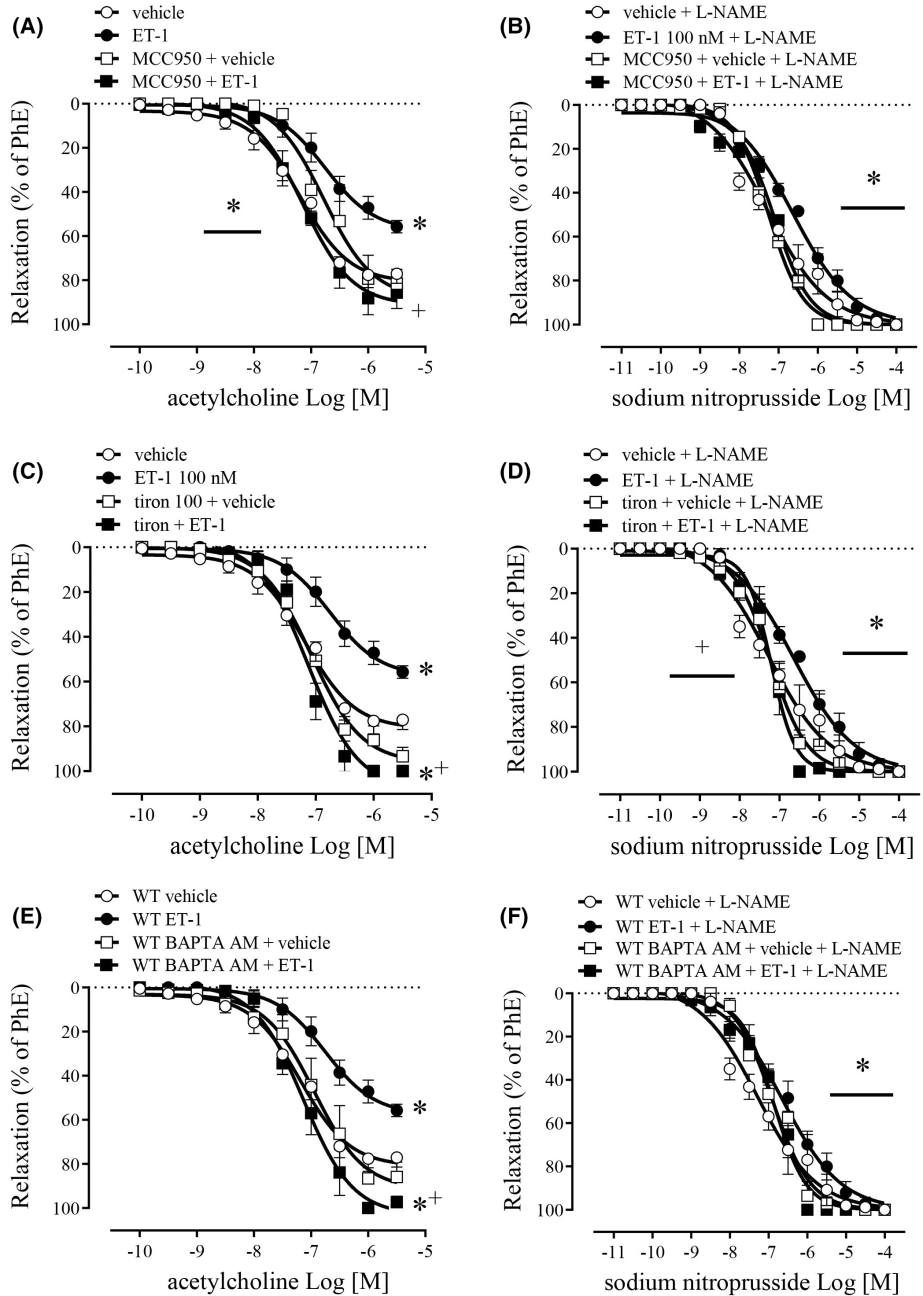
Concentration‐effect curves for ACh (100 pM ‐ 3 μM) (A, C and E) and sodium nitroprusside (10 pM – 100 μM) (B, D and F) in CC strips of WT mice. Tissues were stimulated with ET‐1 (100 nM) in the presence (black squares) or absence (black circles) of MCC950 (1 μM), TIRON (100 μM), BAPTA‐AM (20 μM) or vehicle in the presence (white squares) or absence (white circles) of MCC950 (1 μM), TIRON (100 μM), BAPTA‐AM (20 μM). Data represent the mean ± SEM values of the % of relaxation. *p < 0.05 compared to vehicle group. ^+^ *p* < 0.05 compared ET‐1. *n* = 5–6. The comparison of pEC_50_ and Emax parameters was performed by two‐way anova followed by Tukey post‐test

Endothelin‐1100 nM decreased the endothelium‐dependent relaxation, shifting concentration‐response curves for ACh to the right (Figure 2A, Table 1). ET‐1 also reduced the maximal effect produced by ACh and SNP (Figure 2A,B, Table 1). MCC950 prevented the impairment promoted by ET‐1. Additionally, MCC950 slightly shifted the ACh concentration‐response curve to the right in the vehicle‐treated CC (Figure 2A, Table 1). The values of pEC_50_ and Emax for ACh and SNP are described in Table 1.

**TABLE 1:** Values of Emax and pEC50 for the acetylcholine and sodium nitroprusside concentration‐effect curves in CC of mice

Drug	Vehicle	ET‐1	MCC950 + vehicle	MCC950 + ET‐1
Pharmacological inhibition of NLRP3 on ET‐1‐induced impairment of the CC reactivity				
ACh				
pEC_50_	7.31 ± 0.14	6.98 ± 0.23	6.73 ± 0.14*	7.20 ± 0.08
Emax (%)	77.07 ± 4.43	55.72 ± 2.73*	83.03 ± 5.50	88.16 ± 4.89^+^
SNP				
pEC_50_	7.32 ± 0.14	6.69 ± 0.11*	7.20 ± 0.12	7.06 ± 0.01
Emax (%)	100 ± 3.24	100 ± 1.72	100 ± 1.21	100 ± 1.40

Drug	Vehicle	ET‐1	BQ123 + vehicle	BQ123 + ET‐1
ET_A_ receptor antagonism on ET‐1‐induced impairment in the CC reactivity				
ACh				
pEC_50_	7.31 ± 0.14	6.98 ± 0.23	6.74 ± 0.12	7.52 ± 0.13^#^
Emax	77.07 ± 4.43	55.72 ± 2.73*	100 ± 6.67*	91.04 ± 4.90*^+^
SNP				
pEC_50_	7.32 ± 0.14	6.69 ± 0.11*	7.07 ± 0.09*	8.00 ± 0.13*^#+^
Emax	100 ± 3.24	100 ± 1.72	100 ± 6.37	100 ± 2.88

Drug	Vehicle	ET‐1	BQ788 + vehicle	BQ788 + ET‐1
ET_B_ receptor antagonism on ET‐1‐induced impairment in the CC reactivity				
ACh				
pEC_50_	7.31 ± 0.14	6.98 ± 0.23	6.90 ± 0.11	7.57 ± 0.09^#+^
Emax	77.07 ± 4.43	55.72 ± 2.73*	94.95 ± 5.37*	100*
SNP				
pEC_50_	7.32 ± 0.14	6.69 ± 0.11*	6.98 ± 0.08	7.44 ± 0.08^#+^
Emax	100 ± 3.24	100 ± 1.72	100 ± 3.32	100 + 2.87

Drug	Vehicle	ET‐1	tiron + vehicle	tiron + ET‐1
ROS scavenging with tiron on ET‐1‐induced impairment of the CC reactivity				
ACh				
pEC_50_	7.31 ± 0.14	6.98 ± 0.23	7.07 ± 0.10	7.13 ± 0.07
Emax	77.07 ± 4.43	55.72 ± 2.73*	96.26 ± 4.47*	100 ± 3.51*^+^
SNP				
pEC_50_	7.32 ± 0.14	6.69 ± 0.11*	7.22 ± 0.07	7.21 ± 0.04^+^
Emax	100 ± 3.24	100 ± 1.72	100 ± 1.17	100 ± 1.72

Drug	Vehicle	ET‐1	BAPTA AM + vehicle	BAPTA AM + ET‐1
Ca^2+^ chelation with BAPTA AM on ET‐1‐induced impairment of the CC reactivity				
ACh				
pEC_50_	7.31 ± 0.14	6.98 ± 0.23	6.94 ± 0.13	7.13 ± 0.08
Emax	77.07 ± 4.43	55.72 ± 2.73*	91.46 ± 5.90	100 ± 3.75*^+^
SNP				
pEC_50_	7.32 ± 0.14	6.69 ± 0.11*	6.90 ± 0.09*	6.86 ± 0.05*
Emax	100 ± 3.24	100 ± 1.72	100 ± 1.72	100 ± 1.72

*Note*: Curves were performed after ET‐1 incubation and in the presence or absence of MCC950, Tiron, and BAPTA AM. Values are mean ± SEM (*n* = 5–6 in each group). *p < 0.05 compared to vehicle group. ^#^ *p* < 0.05 compared to MCC950, tiron or BAPTA AM, BQ123 or BQ888 + vehicle. ^+^ *p* < 0.05 compared ET‐1. The comparison of pEC_50_ and Emax parameters was performed by two‐way anova followed by Tukey.

Tiron abolished the deleterious effect of ET‐1 on the CC relaxation in response to ACh (Figure 2C) and SNP (Figure 2D). The values of pEC_50_ and Emax produced by ACh and SNP are described in Table 1.

BAPTA AM entirely reversed the impairment caused by ET‐1 of ACh‐ (Figure 2E) and SNP‐induced (Figure 2F) relaxation. The values of pEC_50_ and Emax for ACh and SNP are described in Table 1.

**FIGURE 3:**
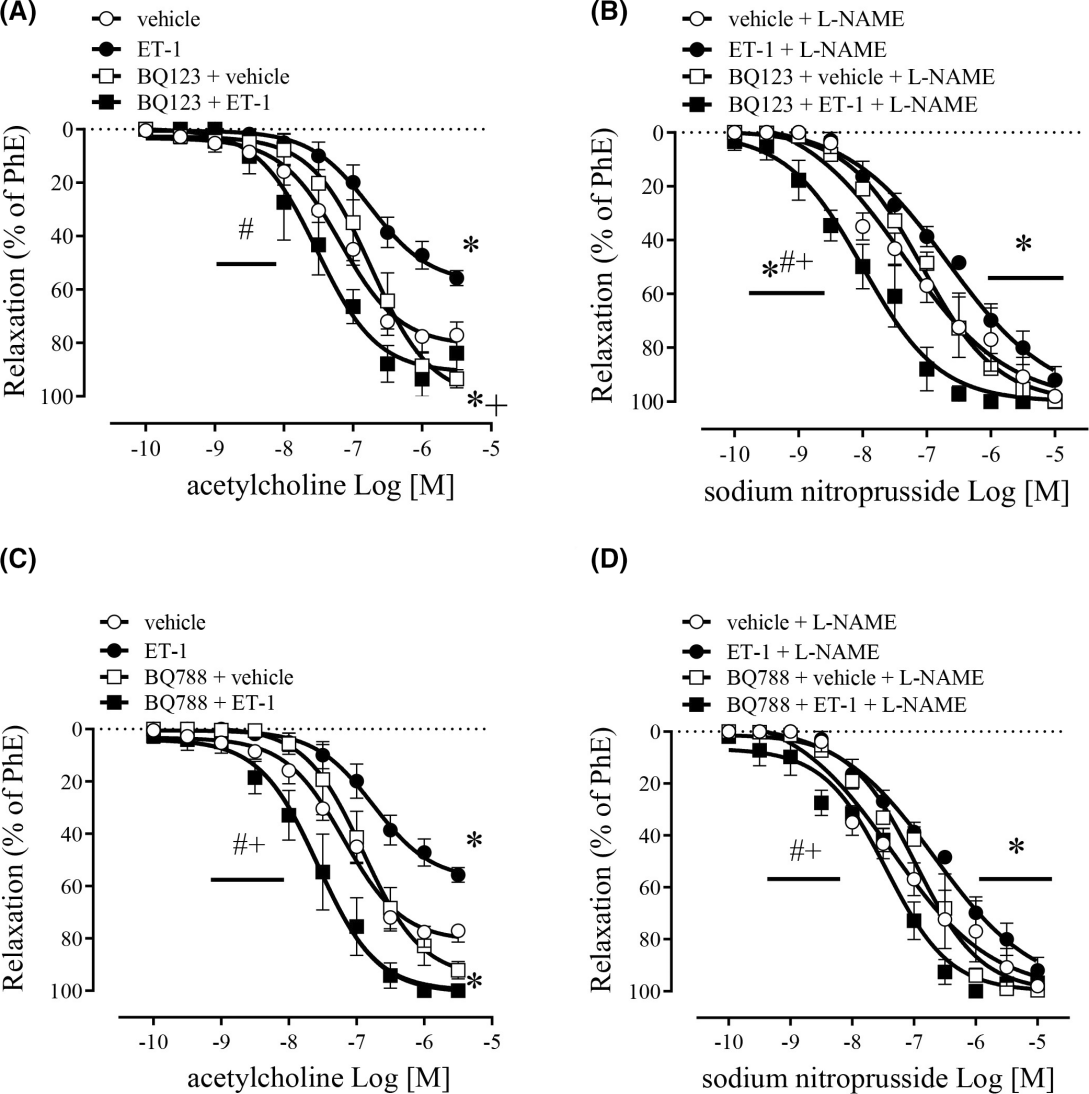
Concentration‐effect curves for ACh (100 pM – 3 μM) (A and C) and sodium nitroprusside (10 pM – 100 μM) (B and D) in CC strips of WT mice incubated with BQ123 or BQ788 (1 μM) followed by ET‐1 (100 nM) (black squares), BQ123 or BQ788 (1 μM) followed by vehicle (white squares), ET‐1 (100 nM) (black circles) or vehicle (white circles). Data represent the mean ± SEM values of the % of relaxation. *p < 0.05 compared to vehicle group. ^#^ *p* < 0.05 compared to BQ123, or BQ888 + vehicle. ^+^ *p* < 0.05 compared ET‐1. *n* = 5–6. The comparison of pEC_50_ and Emax parameters was performed by two‐way anova followed by Tukey post‐test

BQ123 prevented ET‐1‐induced reduction of ACh‐induced CC relaxation (Figure 3A) and of sodium nitroprusside relaxation (Figure 3B). Noteworthy, BQ123 prevented the deleterious effect of ET‐1 and provoked a leftward‐shift in SNP concentration‐effect curves in mice CC (Figure 3B). BQ788 also significantly prevented the effects of ET‐1 on ACh‐ (Figure 3C) and SNP‐induced relaxation (Figure 3D). The values of pEC_50_ and Emax for ACh and SNP are described in Table 1.

The relaxation of CC strips to ACh (Figure S1A) and SNP (Figure S1B) was impaired by ET‐1 in WT mice. NLRP3^−/−^ mice displayed impaired CC relaxation to ACh when compared to the control group. Nevertheless, NLRP3 genetic deletion prevented the ET‐1‐induced impairment of ACh (Figure S1A) and SNP (Figure S1B) relaxation. The values of pEC_50_ and Emax induced by ACh and SNP are described in Table S1.

Genetic deletion of caspase‐1/11 also prevented the effects of ET‐1 on ACh and SNP relaxation (Figures S2A and S2B). However, relaxations induced by ACh and SNP were impaired in CC from caspase‐1/11^−/−^ mice when compared to those in CC from wild‐type mice (Figures S2A and S2B). The values of pEC_50_ and Emax induced by ACh and SNP are described in Table S1.

### 3.3 Effect of endothelin‐1 and NLRP3 inhibition on caspase activity

**FIGURE 4:**
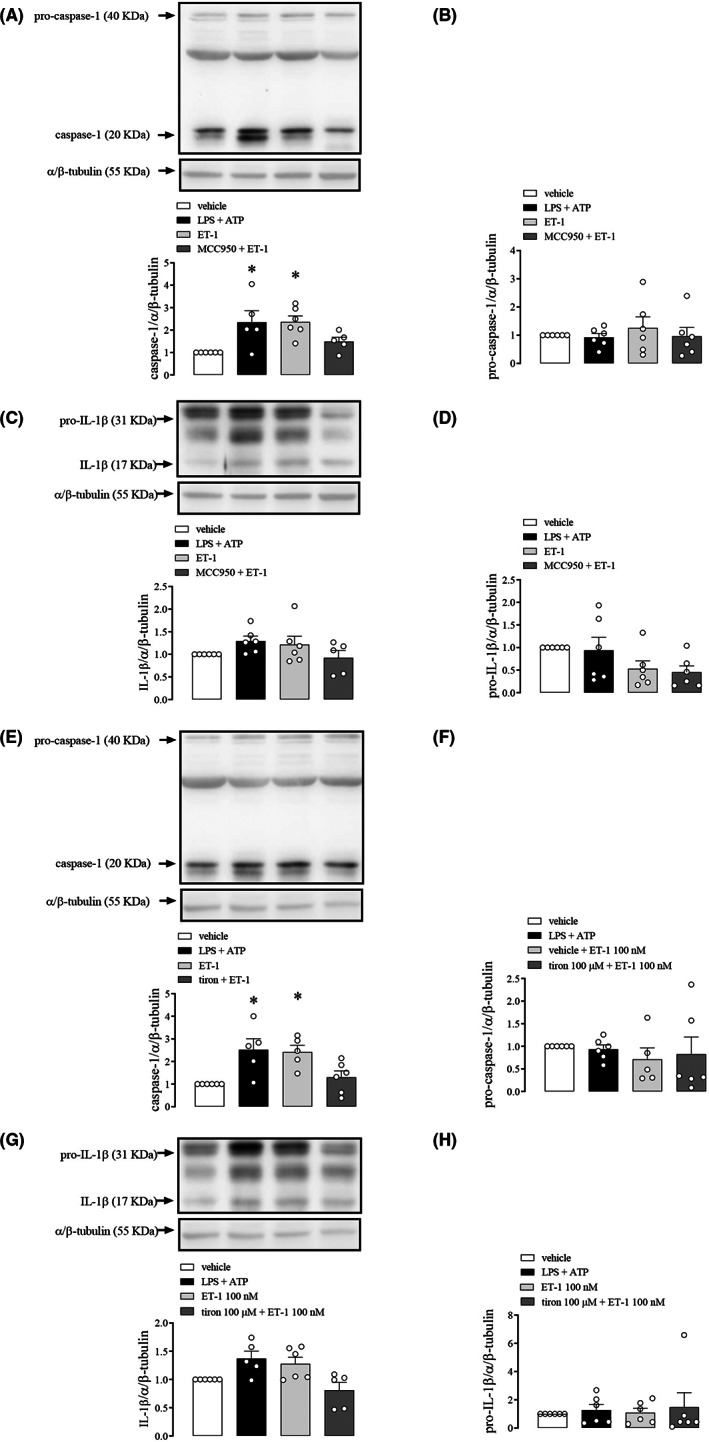
Densitometric analysis of caspase‐1 (A and E), pro‐caspase‐1 (B and F), IL‐1β (C and G) and pro‐IL‐1β (D and H) in WT mice CC strips incubated with LPS + ATP (1 μg/ml + 2 mM) (black bars), ET‐1 (100 nM) (light grey bars), and MCC950 (1 μM) or TIRON (100 μM) followed by ET‐1 (100 nM) (dark grey bars) or vehicle (white bars). The expression of α/β‐tubulin was determined and used as the internal control. The bars represent the mean ± SEM values of protein expression. *p < 0.05 compared to vehicle group. *n* = 6. The comparison of protein expression was performed by one‐way anova followed by Tukey post‐test. Caspase‐1 and IL‐1β proteins were blotted in the same membrane. Therefore, both proteins share the same α/β‐tubulin image band for quantification analysis as an internal control and for representative image between (A and C) and also between the (E and G)

NLRP3 pharmacological inhibition with MCC950 prevented increased caspase‐1 activation (Figure 4A). Pro‐caspase‐1 expression was not altered in CC treated with LPS + ATP, ET‐1 or MCC950 + ET‐1 (Figure 4B). The expression of IL‐1β or pro‐IL‐1β was not significantly altered (Figure 4C,D).

**FIGURE 5:**
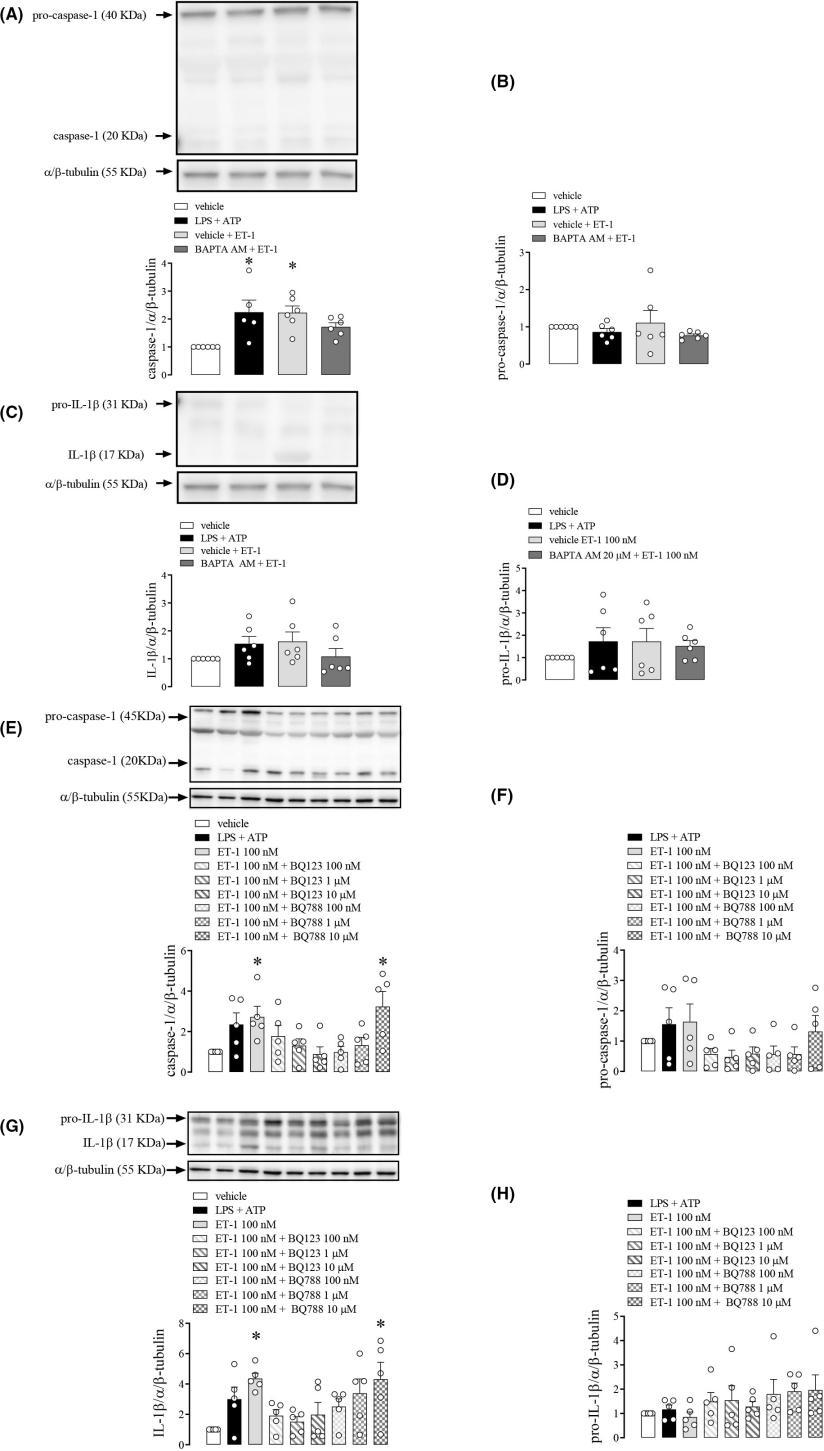
Densitometric analysis of caspase‐1 (A and E), pro‐caspase‐1 (B and F), IL‐1β (C and G) and pro‐IL‐1β (D and H) in WT mice CC strips incubated with LPS + ATP (1 μg.ml^−1^ + 2 mM) (black bars), ET‐1 (100 nM) (light grey bars), BAPTA AM (100 μM), BQ123 (scratched) and BQ788 (chess) 100 nM (light grey), 1 (medium grey) or 10 μM (dark grey) followed by ET‐1 (100 nM) or vehicle (white bars). The expression of α/β‐tubulin was determined and used as the internal control. The bars represent the mean ± SEM values of protein expression. *p < 0.05 compared to WT group. *n* = 6. The comparison of protein expression was performed by anova followed by Tukey. Caspase‐1 and IL‐1β proteins were blotted in the same membrane. Therefore, both proteins share the same α/β‐tubulin image band for quantification analysis as an internal control and for representative image between (A and C) and also between the (E and G)

Tiron inhibited the increase in caspase‐1 expression evoked by ET‐1 (Figure 4E) in mice CC. However, no significant changes were observed in pro‐caspase1 (Figure 4F), IL‐1β (Figure 4G), and pro‐IL‐1β expression (Figure 4H) (original western blot membranes for MCC950 and tiron are in Figure S7). Additionally, BAPTA AM significantly reduced caspase‐1 activation (Figure 5A). Nevertheless, no significant changes were observed in pro‐caspase1 (Figure 5B), IL‐1β (Figure 5C) and pro‐IL‐1β expression (Figure 5D) (original western blot membranes for BAPTA AM are in Figure S8).

BQ123 reduced, in a dose‐dependent manner, the increase in caspase‐1 (Figure 5E) and IL‐1β (Figure 5G) expressions induced by ET‐1 in mice CC. However, no significant changes were observed in pro‐caspase1 (Figure 5F) and pro‐IL‐1β expression (Figure 5H).

Interestingly, BQ788 blocked the increase in caspase‐1 and IL‐1β expressions by ET‐1 in CC (Figure 5E,G). However, the blockade by BQ788 only occurred at lower concentrations, and it was reversed in a dose‐dependent manner at higher concentrations (Figure 5E,G) (original western blot membranes for BQ123 and BQ788 are in Figure S6).

**FIGURE 6:**
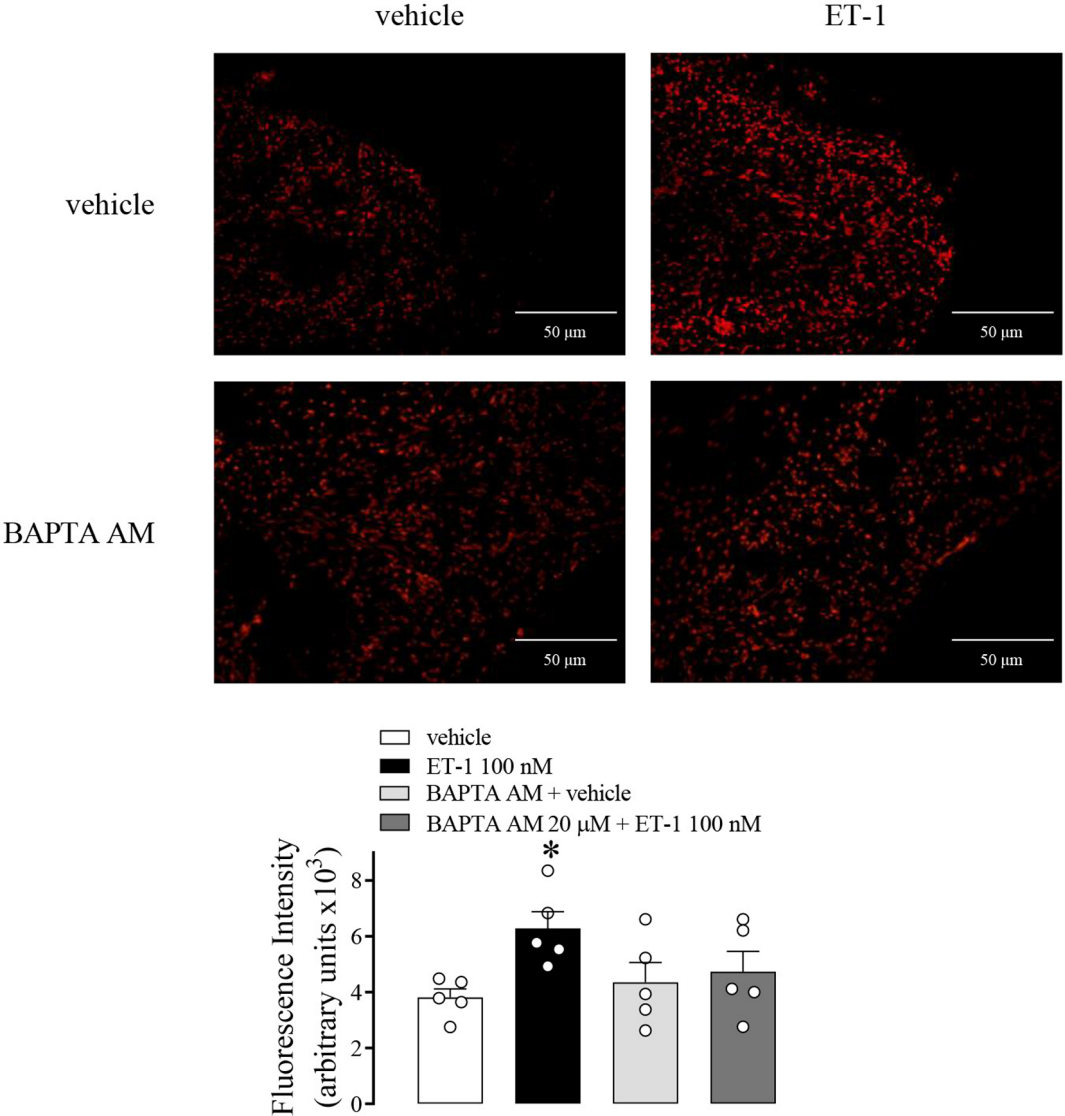
ROS generation in CC measured by DHE in WT mice CC strips incubated with LPS + ATP (1 μg.ml^−1^ + 2 mM) (black bars), ET‐1 (100 nM) (light grey bars), BAPTA AM (100 μM) followed by ET‐1 (100 nM) (dark grey bars) or vehicle (white bars). The bars represent the mean ± SEM values of protein expression. *p < 0.05 compared to vehicle group. *n* = 6. The comparison of ROS generation by DHE was performed by two‐way anova followed by Tukey post‐test

Endothelin‐1 significantly increased ROS generation in CC strips, which was abolished in the presence of a calcium chelator (BAPTA AM) (Figure 6).

Endothelin‐1 induced a significant increase in caspase‐1 expression (Figure S3A), in a concentration‐dependent manner, in CC of WT mice. Although ET‐1 at 100 nM increased caspase‐1, it did not change IL‐1β expression (Figure S3C). No changes in pro‐caspase‐1 (Figure S3B) or pro‐IL‐1β (Figure S3D) expression were observed after ET‐1 stimulation. Interestingly, ET‐1 activated NLRP3 signalling without a prior priming stimulus as in the positive control group, which was primed with LPS for 4 h before the addition of ATP (LPS + ATP). The positive control group and the ET‐1 group displayed similar patterns for caspase‐1 (Figure S3A), pro‐caspase‐1 (Figure S3B), IL‐1β (Figure S3C), and pro‐IL‐1β (Figure S3D) expression (original western blot membranes for ET‐1 are in Figure S5).

NLRP3 genetic deletion prevented activation of caspase‐1 (Figure S4A) induced by ET‐1. However, CC from NLRP3^−/−^ mice have increased basal expression of caspase‐1 (Figure S4A) (original western blot membranes for NLRP3 genetic deletion are in Figure S9).

## 4 DISCUSSION

In our studies, we found that overactivation of NLRP3 impairs the cavernosal function,^35^ and that ET‐1, via ET_A,_ reduces erectile function in DOCA/salt rats.^6^ The novelty of the present study is that ET‐1‐induced erectile dysfunction depends on NLRP3 activation. Moreover, we found that ET_A_ and ET_B_ receptors mediate the activation of NLRP3 by ET‐1 in mouse CC via Ca^2+^‐dependent ROS generation.

Endothelin‐1 plays an essential role in the pathogenesis of several cardiovascular diseases, including erectile dysfunction.^6^ ET‐1 acts as a proinflammatory modulator, releasing cytokines such as IL‐1β, IL‐6, and TNF‐α.^9^, ^10^, ^45^, ^46^ NLRP3 is a crucial modulator of the innate immune response. Vascular cells detect and respond to damage‐associated molecular patterns (DAMPs) or pathogen‐associated molecular patterns (PAMPs) via TLRs and NLRs. NLRP3 leads to the release of cytokines and chemokines.^40^, ^47^, ^48^

Endothelin‐1 induces IL‐1β release^9^ by a mechanism involving direct stimulation of NF‐*κ*B.^49^ Moreover, the DOCA/salt hypertension model exhibits increased caspase‐1 and IL‐1β activity in the kidney,^50^ and it is characterized by ET‐1 overexpression.^6^, ^51^ As observed in the present study, ET‐1 does not need previous stimulation with LPS to increase pro‐caspase‐1 and pro‐IL‐1β, and the increased caspase‐1 and IL‐1β are in agreement with direct stimulation of NF‐*κ*B.

NF‐*κ*B modulates the proinflammatory activity of the ET‐1 in lung^15^ or liver tissues.^14^ NF‐*κ*B is the first signal for the NLRP3 activation.^33^ Additionally, ET‐1 induces IL‐1β release in monocytes^9^ and endothelial cells.^45^ IL‐1β and IL‐18 are the main products of NLRP3 activation.^14^ Indeed, the pharmacological inhibition or genetic deletion of NLRP3 prevented caspase‐1 activation produced by ET‐1 in the CC, hence suggesting that NLRP3 has an important role in ET‐1‐induced caspase‐1 activity in the CC.

Increased NLRP3‐induced IL‐1β release impairs the vascular function in aldosterone‐infused mice.^40^ Also, NLRP3 is essential for hypertension development in the DOCA/salt model,^50^ mainly due to increased leukocyte recruitment and consequent renal fibrosis.^52^ In previous studies, we also found that overactivation of NLRP3 impairs endothelium‐dependent relaxation in CC (Fais et al., 2019). In agreement with these findings, NLRP3 inhibition in the present study prevented the impairment promoted by ET‐1 in endothelium‐dependent and independent relaxation of CC, which translated into a beneficial effect on erectile function. A limitation of the present findings is that the NLRP3 inhibitor, MCC950, decreased acetylcholine relaxation in the CC from vehicle‐treated mice, a finding similar to previous studies.^35^ However, further studies are required to clarify whether this effect can be ascribed to NLRP3 or that MCC950 may also have NLRP3‐independent effects interfering with endothelium‐dependent relaxations.

Increased ROS generation is a second signal for the NLRP3 activation.^33^ Moreover, ET‐1 increases ROS generation in rat liver^14^ and lung,^15^ releasing proinflammatory cytokines via NF‐κB. ET‐1 contributes to sickle cell nephropathy in humanized homozygous Hb mice, specifically via ET_A_‐induced increase in NADPH oxidase subunits.^53^ Furthermore, ET‐1 increases ROS generation in obese Zucker rats penile smooth muscle cells.^8^ In addition, ET‐1 increases mitochondrial ROS generation in ventricular myocytes^54^ through the proton influx, mediated by Na^(+)^/H^(+)^ exchangers, possibly through increased superoxide anion (O_2_^−^) generation.^55^ In agreement with these previous findings, a ROS scavenger prevented the ET‐1‐induced caspase‐1 activation in rat CC.^8^, ^54^, ^55^

There is a cross‐talk between ROS and Ca^2+^ signalling.^30^ Incubation with an intracellular Ca^2+^ chelator prevented the oxidative stress induced by ET‐1 in CC. In the present study, ET‐1 increased ROS generation in mice CC. Additionally, BAPTA AM inhibited the ET‐1‐induced caspase‐1 increase suggesting that Ca^2+^ contributes to the activation of NLRP3.

Endothelin‐1 induces ET_A_‐dependent superoxide anion generation and impairs the penile artery function relaxation in obese rats. However, ET_B_ also importantly modulates ROS generation.^8^ In the present manuscript, both ET_A_ and ET_B_ receptors activated caspase‐1. In addition, tiron prevented the impairment promoted by ET‐1 on ACh‐ and SNP‐induced relaxation. Thus, ET_A_ and ET_B_ receptors differentially modulate the NLRP3 activity, which may explain unsuccessful results with ET‐1 antagonists in clinical trials.

Oxidative stress and increased Ca^2+^ influx are a second signal for the NLRP3 inflammasome formation.^28^, ^29^, ^56^ Moreover, ET‐1‐induced ROS generation depends on Ca^2+^ influx in pulmonary vascular smooth muscle^22^ and mesangial cells.^23^ Also, the Ca^2+^ chelator inhibited the CC relaxation impairment promoted by ET‐1. These findings suggest that ROS and Ca^2+^ influx contribute to NLRP3 activation and the impairment of NO‐mediated relaxation in rat CC stimulated with ET‐1. ET‐1 also modulates nerve response, which can modulate the cardiovascular function.^24^, ^25^ However, our present study has a limitation that is focused only on the vascular actions of ET‐1.

## 5 CONCLUSION AND PERSPECTIVES

In summary, our study shows that NLRP3 mediates the impairment in erectile function and CC relaxation induced by ET‐1. These changes are highly dependent on increased ROS generation since its inhibition reduced caspase‐1 and restored the CC reactivity. Additionally, ET‐1‐induced oxidative stress depends on Ca^2+^. Also, ET‐1‐induced NLRP3 activation is influenced by both ET_A_ and ET_B_ receptors. Therefore, ET‐1‐induced NLRP3 activation may represent a novel target to modulate the erectile function. Thus, our findings suggest that both the endothelin pathway and activation of NLRP3 play a role in erectile dysfunction and impaired CC relaxation. Several studies have shown no effect of endothelin receptor antagonists on erectile dysfunction.^5^, ^6^, ^57^ However, as we observed in this manuscript, ET_A_ and ET_B_ play different roles in NLRP3 activation end erectile function modulation. This could partially explain why those studies failed to show the effect of ET receptor antagonists on erectile function. A previous study showed that NLRP3 modulates macrophage recruitment and vascular impairment in aldosterone‐infused mice. Additionally, we demonstrated that NLRP3 modulates the CC function.^35^, ^40^ The present study shows that activation of endothelin receptors leads to NLRP3 activation, followed by CC function impairment. Therefore, further research will be required to elucidate whether early intervention may prevent the activation of the endothelin pathway and NLRP3 in patients with risk factors for erectile dysfunction.

## AUTHOR CONTRIBUTIONS

**Rafael Sobrano Fais:** Conceptualization (equal); data curation (equal); formal analysis (equal); investigation (equal); methodology (equal); project administration (equal); writing – original draft (equal); writing – review and editing (equal). **Rafael Menezes da Costa:** Conceptualization (equal); data curation (equal); writing – original draft (equal). **Allan Carvalho Mendes:** Methodology (equal). **Fabiola Mestriner:** Methodology (equal). **Simon Gabriel Comerma‐Steffensen:** Data curation (equal); formal analysis (equal); methodology (equal). **Rita C. Tostes:** Conceptualization (equal); data curation (equal); formal analysis (equal); funding acquisition (equal); supervision (equal); validation (equal); visualization (equal); writing – original draft (equal); writing – review and editing (equal). **Ulf Simonsen:** Conceptualization (equal); data curation (equal); formal analysis (equal); funding acquisition (equal); supervision (equal); validation (equal); visualization (equal); writing – original draft (equal); writing – review and editing (equal). **Fernando Silva Carneiro:** Conceptualization (equal); data curation (equal); formal analysis (equal); funding acquisition (equal); supervision (equal); validation (equal); visualization (equal); writing – original draft (equal); writing – review and editing (equal).

## CONFLICT OF INTEREST

The authors declare no conflict of interest.

## Supporting information


Appendix S1

Appendix S2

## Data Availability

The data supporting this study's findings are available from the corresponding author upon reasonable request. Some data may not be made available because of privacy or ethical restrictions.
