# Supplementary material for: NLRP3 activation contributes to endothelin‐1‐induced erectile dysfunction
Source: J Cell Mol Med. 2022 Dec 14;27(1):1–14. doi: 10.1111/jcmm.17463 (PMC9806301; doi:10.1111/jcmm.17463)
Supplement: Supplementary file 1 — Appendix S1 [file JCMM-27-1-s002.pdf]

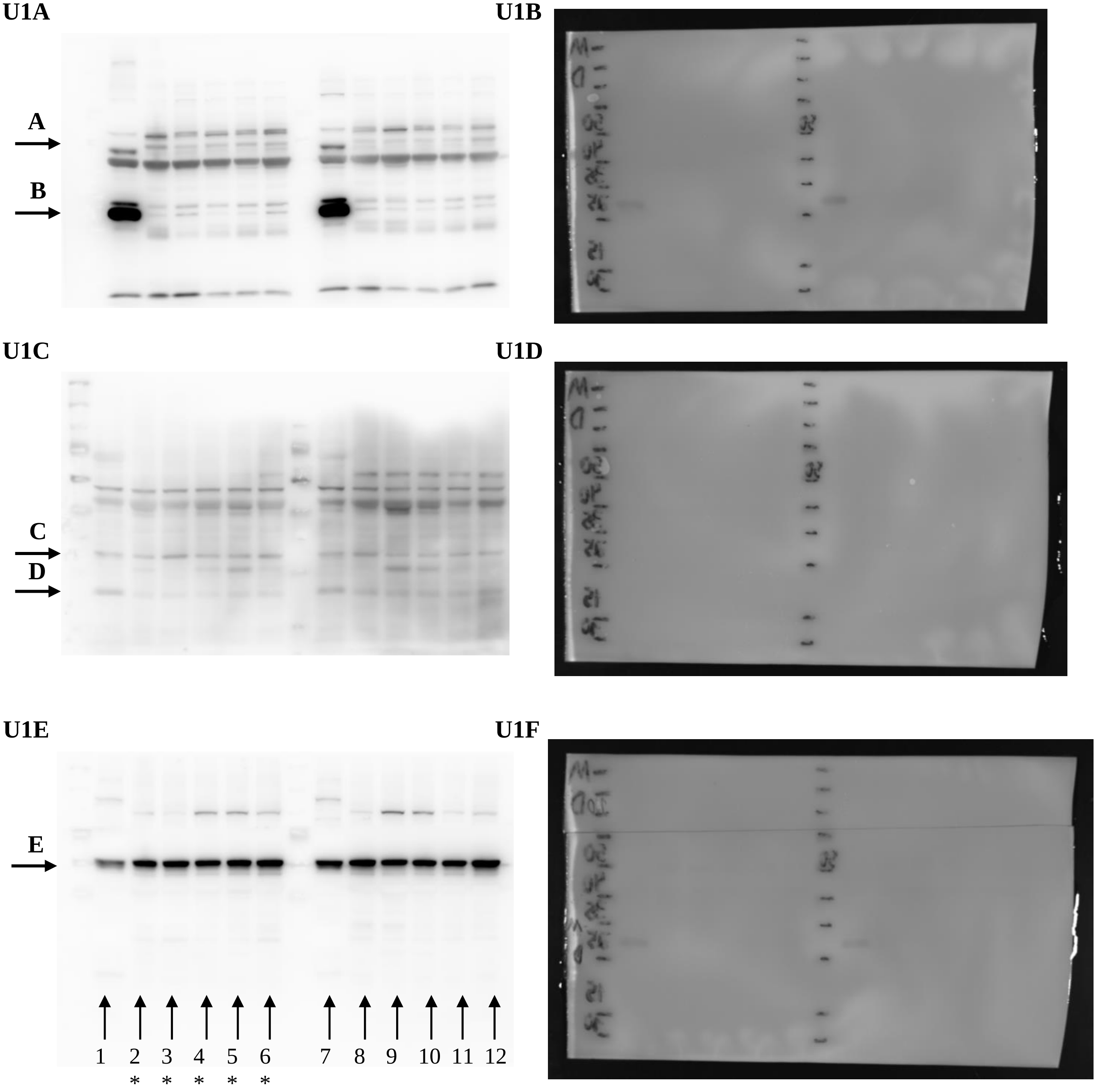

A: pro-caspase-1; **B**: caspase-1; **C**: pro-IL-1β; **D**: IL-1β; **E**: α/β-tubulin

Lane 1: Macrophages LPS+ATP
Lane 2: CC vehicle
Lane 3: CC LPS+ATP
Lane 4: CC ET-1 1 nM
Lane 5: CC ET-1 10 nM
Lane 6: CC ET-1 100 nM
Lane 7: CC Macrophages lysate LPS+ATP
Lane 8: CC vehicle
Lane CC 9: LPS+ATP
Lane CC 10: ET-1 1 nM
Lane CC 11: ET-1 10 nM
Lane CC 12: ET-1 100 nM

Figure U1. Original membrane showing the Figure **S3** pro-caspase and caspase-1 bands obtained by chemiluminescence **(A)**, the same membrane visualized by epi-luminescence **(B)**. Original membrane showing the pro-IL-1β and IL-1β bands obtained by chemiluminescence **(C)**, the same membrane visualized by epi-luminescence **(D)**. Original membrane showing the α-tubulin bands obtained by chemiluminescence **(C)**, the same membrane visualized by epi-luminescence **(D)**. * Represents the bands used for the representative images.

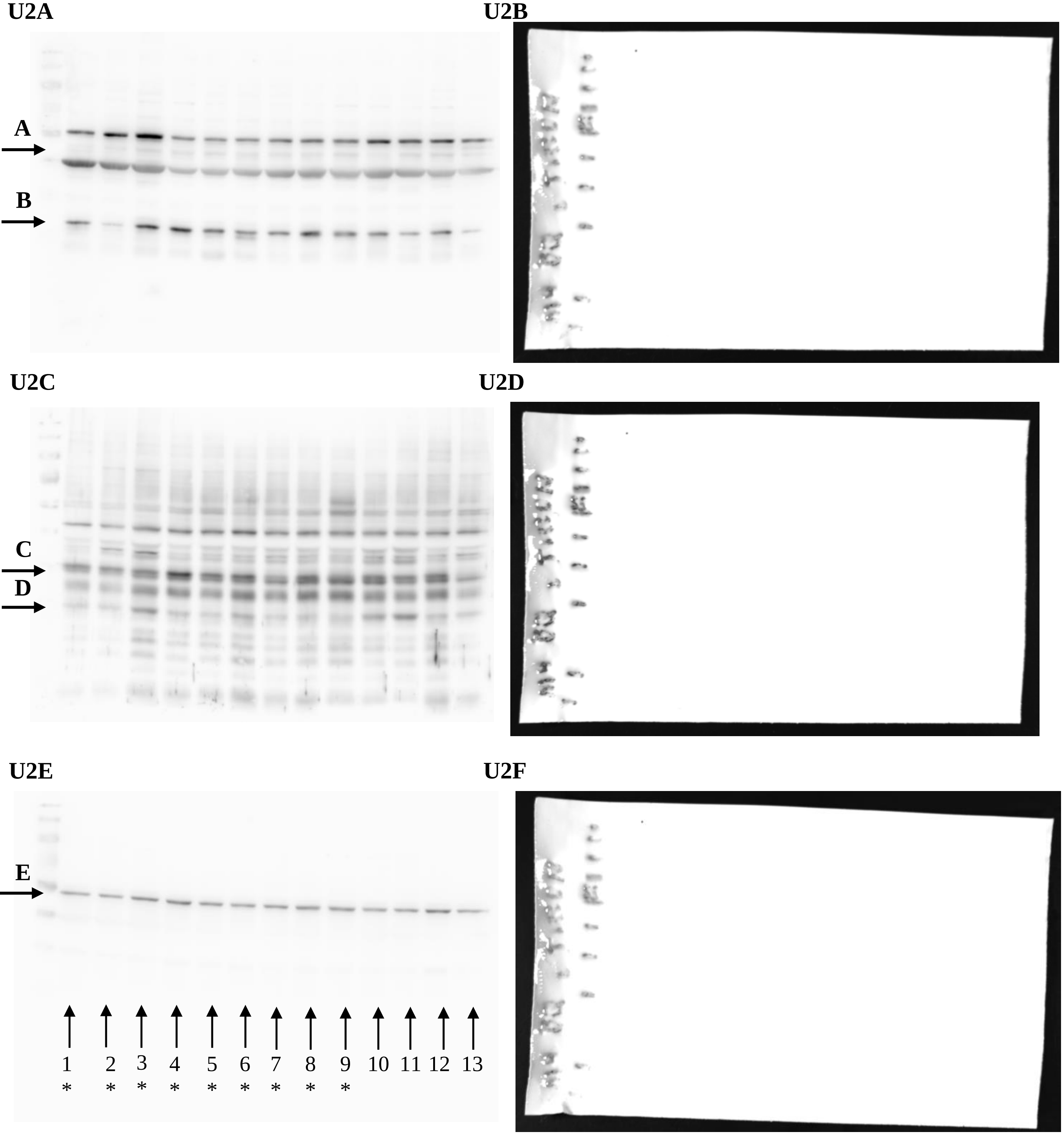

A: pro-caspase-1; **B**: caspase-1; **C**: pro-IL-1β; **D**: IL-1β; **E**: α/β-tubulin

Lane 1: vehicle
Lane 2: LPS+AT
Lane 3: ET-1 100 nM
Lane 4: ET-1 100 nM + BQ123 100 nM
Lane 5: ET-1 100 nM + BQ123 1 µM
Lane 6: ET-1 100 nM + BQ123 10 µM
Lane 7: ET-1 100 nM + BQ788 100nM
Lane 8: ET-1 100 nM + BQ788 1 µM
Lane 9: ET-1 100 nM + BQ788 10 µM
Lane 10: vehicle
Lane 11: LPS+ATP
Lane 12: ET-1 100 nM
Lane 13: ET-1 100 nM + tiron 1 µM

Figure U2 Original membrane showing the Figure **7** pro-caspase and caspase-1 bands obtained by chemiluminescence **(A)**, the same membrane visualized by epi-luminescence **(B)**. Original membrane showing the pro-IL-1β and IL-1β bands obtained by chemiluminescence **(C)**, the same membrane visualized by epi-luminescence **(D)**. Original membrane showing the α-tubulin bands obtained by chemiluminescence **(C)**, the same membrane visualized by epi-luminescence **(D**). * Represents the bands used for the representative images.

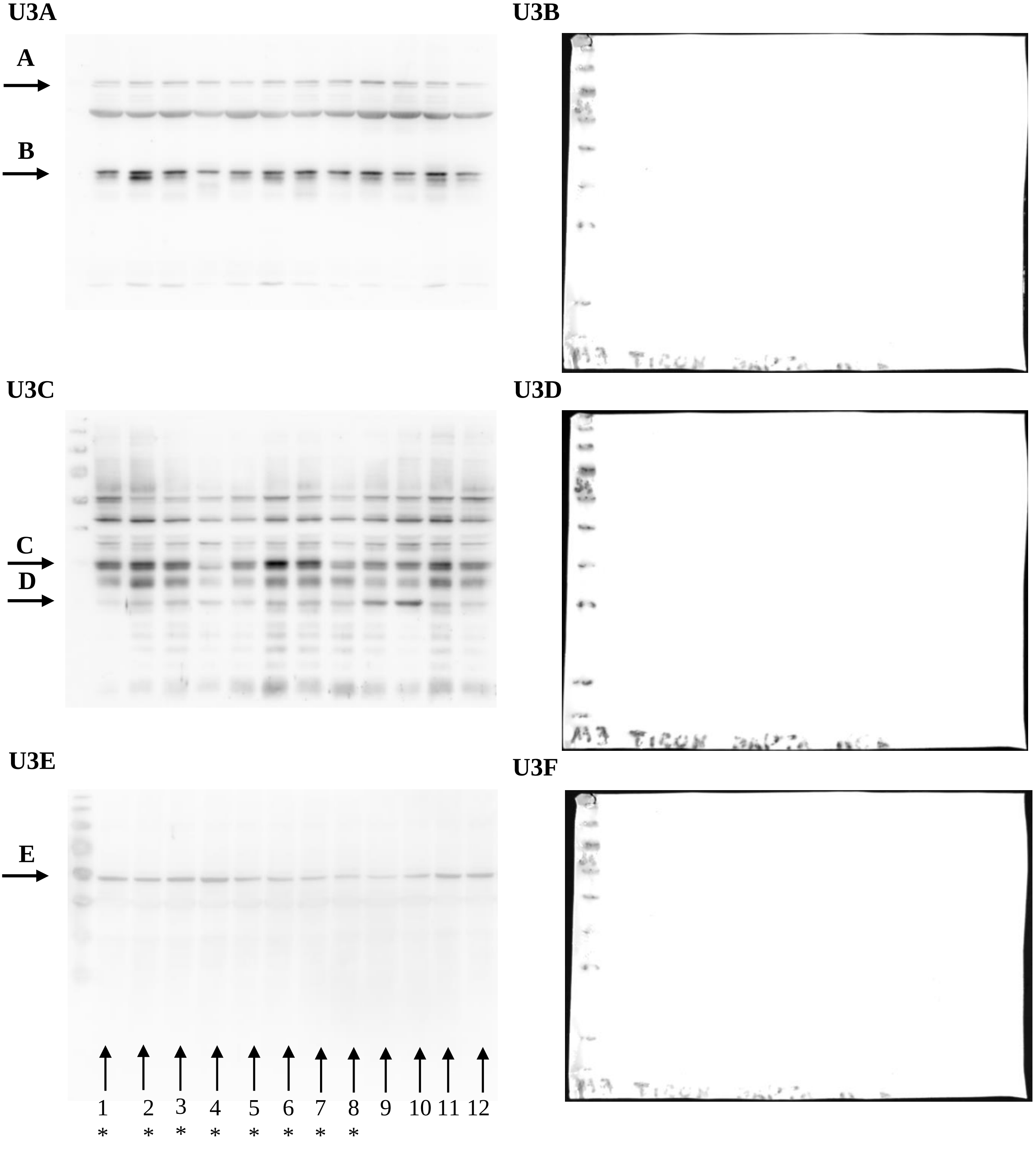

A: pro-caspase-1; **B**: caspase-1; **C**: pro-IL-1β; **D**: IL-1β; **E**: α/β-tubulin

Lane 1: vehicle
Lane 2: LPS+ATP
Lane 3: ET-1 100 nM
Lane 4: MCC950 1 µM + ET-1 100 nM

Lane 5: vehicle
Lane 6: LPS+ATP
Lane 7: ET-1 100 nM
Lane 8: tiron 100 mM + ET-1 100 nM

Lane 9: vehicle
Lane 10: LPS+ATP
Lane 11: ET-1 100 nM
Lane 12: BAPTA AM 20 µM + ET-1 100 nM

Figure U3. Original membrane showing the Figure **6** and **8** pro-caspase and caspase-1 bands obtained by chemiluminescence **(A)**, the same membrane visualized by epi-luminescence **(B)**. Original membrane showing the pro-IL-1β and IL-1β bands obtained by chemiluminescence **(C)**, the same membrane visualized by epi-luminescence **(D)**. Original membrane showing the α-tubulin bands obtained by chemiluminescence **(C)**, the same membrane visualized by epi-luminescence **(D)**. * Represents the bands used for the representative images.

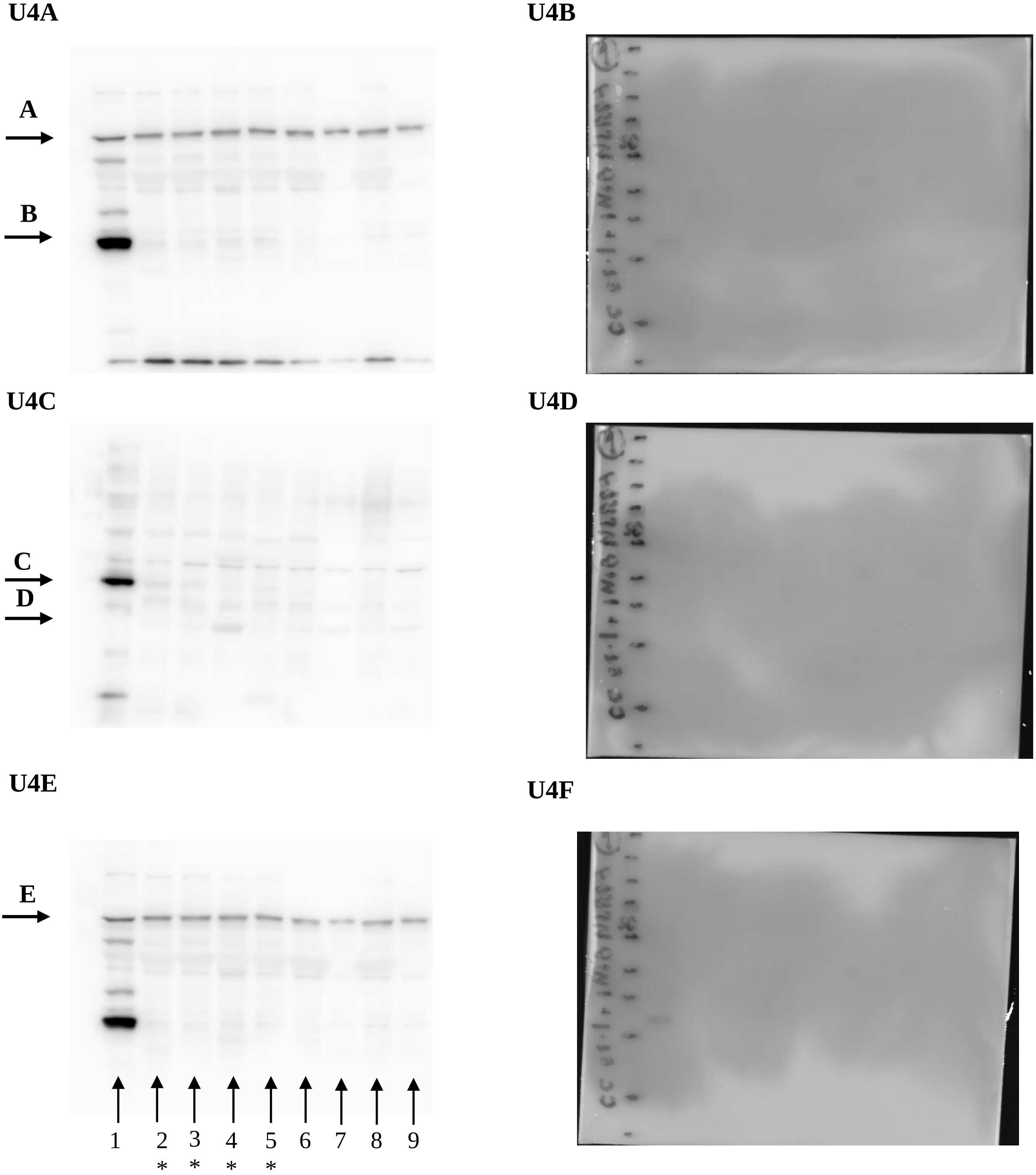

A: pro-caspase-1; **B**: caspase-1; **C**: pro-IL-1β; **D**: IL-1β; **E**: α/β-tubulin

Lane 1: Macrophages LPS + ATP
Lane 2: vehicle
Lane 3: LPS+ATP
Lane 4: ET-1 100 nM
Lane 5: BAPTA AM 20 µM + ET-1 100 nM
Lane 6: MCC950 1 µM + ET-1 100 nM
Lane 7: tiron 100 mM + ET-1 100 nM
Lane 8: potassium cloride 3 mM + ET-1 100 nM
Lane 9: caffeic accid phenil ester + ET-1 100 nM

Figure U4. Original membrane showing the Figure **9** pro-caspase and caspase-1 bands obtained by chemiluminescence **(A)**, the same membrane visualized by epi-luminescence **(B)**. Original membrane showing the pro-IL-1β and IL-1β bands obtained by chemiluminescence **(C)**, the same membrane visualized by epi-luminescence **(D)**. Original membrane showing the α-tubulin bands obtained by chemiluminescence **(C)**, the same membrane visualized by epi-luminescence (**D**). * Represents the bands used for the representative images.

U5A

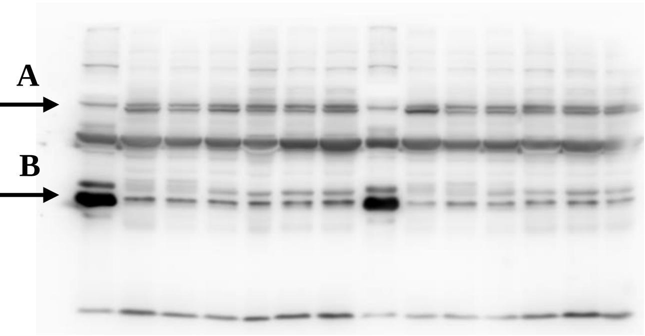

U5B

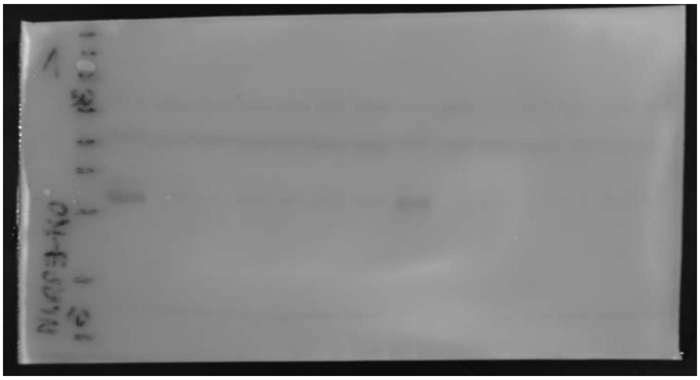

U5C

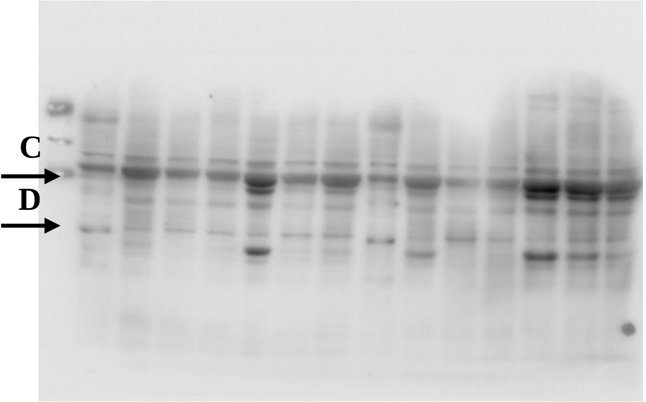

U5D

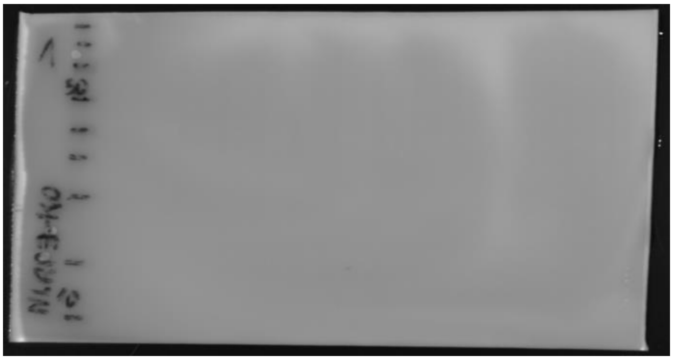

U5E

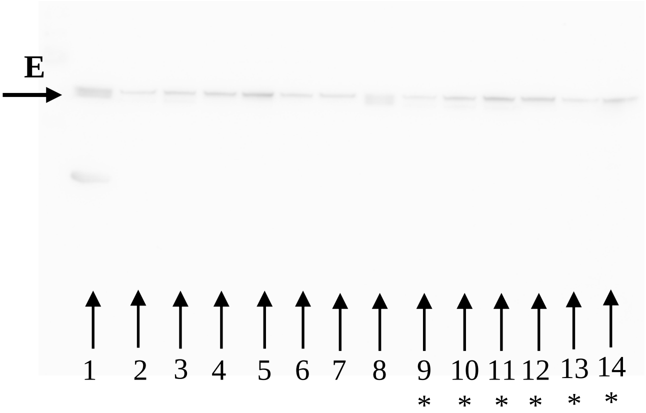

U5F

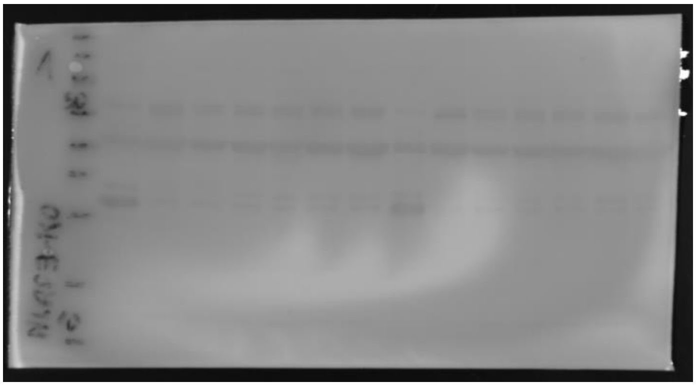

A: pro-caspase-1; **B**: caspase-1; **C**: pro-IL-1β; **D**: IL-1β; **E**: α/β-tubulin

Lane 1: WT macrophages LPS+ATP
Lane 2: WT CC vehicle
Lane 3: WT CC LPS+ATP
Lane 4: WT CC ET-1 100 nM
Lane 5: NLRP3$^{-/-}$ CC vehicle
Lane 6: NLRP3$^{-/-}$ CC LPS + ATP
Lane 7: NLRP3$^{-/-}$ CC ET-1 100 nM
Lane 8: WT macrophages LPS+ATP
Lane 9: WT CC vehicle
Lane 10: WT CC LPS+ATP
Lane 9: WT CC ET-1 100 nM
Lane 10: NLRP3$^{-/-}$ CC vehicle
Lane 11: NLRP3$^{-/-}$ CC LPS + ATP
Lane 12: NLRP3$^{-/-}$ CC ET-1 100 nM

Figure U5. Original membrane showing the Figure **S4** pro-caspase and caspase-1 bands obtained by chemiluminescence **(A)**, the same membrane visualized by epi-luminescence **(B)**. Original membrane showing the pro-IL-1β and IL-1β bands obtained by chemiluminescence **(C)**, the same membrane visualized by epi-luminescence **(D)**. Original membrane showing the α-tubulin bands obtained by chemiluminescence **(C)**, the same membrane visualized by epi-luminescence (**D**). * Represents the bands used for the representative images.
